# Supplementary material for: Faster rehabilitation weight gain during childhood is associated with risk of non-communicable disease in adult survivors of severe acute malnutrition
Source: PLOS Glob Public Health. 2023 Dec 21;3(12):e0002698. doi: 10.1371/journal.pgph.0002698 (PMC10734994; doi:10.1371/journal.pgph.0002698)
Supplement: S1 Table — (DOCX) [file pgph.0002698.s001.docx]

**Supplementary Table 1:** Results of ungrouped linear regressions of rehabilitation weight gain and post-recovery weight and height gain against NCD risk indicators in 273 adult survivors of severe acute malnutrition

| **NCD indicator** | **Weight/**  **height**  **Gain**  **Definition^#^** | **Unadjusted coefficient** | ***p*-value** | **95% CI** | **Adjusted coefficient ^a^** | ***p*-value** | **95% CI** | **Adjusted coefficient ^b^** | ***p*-value** | **95% CI** |
| --- | --- | --- | --- | --- | --- | --- | --- | --- | --- | --- |
| **Systolic BP (mmHg)** | **1** | -13 | 0.6 | -64, 38 | -16 | 0.5 | -66, 33 | -19 | 0.5 | -68, 31 |
|  | **2** | -0.1 | 0.5 | -0.5, 0.2 | -0.1 | 0.7 | -0.5, 0.3 | -0.2 | 0.3 | -0.6, 0.2 |
|  | **3** | -0.01 | 0.8 | -0.06, 0.05 | -0.02 | 0.5 | -0.07, 0.04 | -0.01 | 0.6 | -0.07, 0.04 |
|  | **4** | -10 | 0.5 | -36, 16 | -9 | 0.5 | -35, 17 | -12.9 | 0.4 | -40, 15 |
|  | **5** | -1 | 0.2 | -0.3, 0.07 | -0.1 | 0.3 | -0.3, 0.1 | -0.1 | 0.2 | -0.3, 0.07 |
|  | **6** | -5 | 0.7 | -35, 25 | -5 | 0.8 | -36, 27 | -3 | 0.9 | -35, 29 |
| **Diastolic BP (mmHg)** | **1** | 25 | 0.3 | -23, 72 | 22 | 0.3 | -24, 68 | 20 | 0.4 | -26, 66 |
|  | **2** | 0.1 | 0.7 | -0.3, 0.4 | 0.2 | 0.4 | -0.2, 0.5 | 0.1 | 0.8 | -0.3, 0.4 |
|  | **3** | 0.03 | 0.2 | -0.02, 0.09 | 0.03 | 0.3 | -0.03, 0.08 | 0.03 | 0.3 | -0.02, 0.08 |
|  | **4** | -9.1 | 0.4 | -33, 15 | -4.7 | 0.7 | -28, 19 | -6 | 0.6 | -31, 18 |
|  | **5** | -0.1 | 0.3 | -0.3, 0.07 | -0.1 | 0.5 | -0.2, 0.1 | -0.1 | 0.4 | -0.3, 0.1 |
|  | **6** | 2.7 | 0.9 | -26, 32 | 5.4 | 0.7 | -23, 34 | 8 | 0.6 | -21, 37 |
| **BMI (kg/m^2^)** | **1** | 17 | 0.1 | -4, 38 | 15 | 0.1 | -5, 35 | 15 | 0.1 | -5, 34 |
|  | **2** | 0.04 | 0.6 | -0.1, 0.2 | 0.1 | 0.3 | -0.1, 0.23 | 0.1 | 0.1 | -0.02, 0.28 |
|  | **3** | 0.02 | 0.1 | -0.01, 0.04 | 0.02 | 0.1 | -0.003, 0.04 | 0.02 | 0.2 | -0.01, 0.04 |
|  | **4** | -0.1 | 1.0 | -11, 11 | 5 | 0.3 | -5, 14 | 6.6 | 0.2 | -3, 16 |
|  | **5** | 0.01 | 0.9 | -0.07, 0.09 | 0.03 | 0.4 | -0.04, 0.10 | 0.04 | 0.2 | -0.02, 0.1 |
|  | **6** | 1.7 | 0.8 | -12, 15 | 1.2 | 0.9 | -11, 13 | 2.0 | 0.7 | -10, 14 |
|  | **1** | **55*** | **0.004** | **3, 107** | **53*** | **0.03** | **6, 100** | **53*** | **0.03** | **6, 100** |
|  | **2** | 0.1 | 0.5 | -0.3, 0.5 | 0.3 | 0.2 | -0.1, 0.6 | **0.4*** | **0.02** | **0.1, 0.8** |
|  | **3** | **0.1*** | **0.01** | **0.02, 0.1** | **0.1*** | **0.007** | **0.02, 0.1** | **0.1*** | **0.02** | **0.01, 0.1** |
| **Waist circumference** | **4** | -5 | 0.7 | -32, 22 | 7.0 | 0.5 | -15, 29 | 11 | 0.3 | -12, 34 |
| **(cm)** | **5** | -0.03 | 0.7 | -0.2, 0.2 | 0.03 | 0.7 | -0.1, 0.2 | 0.1 | 0.4 | -0.1, 0.2 |
|  | **6** | 0.5 | 0.7 | -2, 3 | 0.9 | 0.5 | -2, 3 | 0.9 | 0.5 | -2, 3 |
| **Waist-hip ratio** | **1** | 0.1 | 0.5 | -0.2, 0.4 | 0.1 | 0.3 | -0.3, 0.4 | 0.1 | 0.3 | -0.1, 0.4 |
|  | **2** | 0.00004 | 1.0 | -0.002, 0.002 | 0.001 | 0.4 | -0.001, 0.003 | 0.001 | 0.4 | -0.001, 0.004 |
|  | **3** | 0.0002 | 0.3 | -0.0001, 0.0005 | 0.0002 | 0.2 | -0.0001, 0.0005 | 0.0002 | 0.2 | -0.0001, 0.0005 |
|  | **4** | 0.01 | 0.9 | -0.14, 0.16 | 0.02 | 0.8 | -0.13, 0.16 | -0.01 | 0.9 | -0.16, 0.14 |
|  | **5** | -0.0002 | 0.8 | -0.001, 0.001 | 0.00004 | 0.9 | -0.001, 0.001 | -0.0002 | 0.8 | -0.001, 0.001 |
|  | **6** | 0.04 | 0.6 | -0.13, 0.21 | 0.03 | 0.8 | -0.14, 0.18 | 0.02 | 0.8 | -0.14, 0.17 |
| **Lean mass (kg)** | **1** | 14 | 0.5 | -30, 58 | **37*** | **0.02** | **7, 68** | **37*** | **0.02** | **7, 67** |
|  | **2** | 0.1 | 0.7 | -0.28, 0.39 | 0.1 | 0.3 | -0.1, 0.4 | **0.3*** | **0.02** | **0.1, 0.5** |
|  | **3** | 0.04 | 0.1 | -0.01, 0.09 | **0.1*** | **0.006** | **0.01, 0.08** | **0.04*** | **0.02** | **0.01, 0.07** |
|  | **4** | 11 | 0.3 | -9, 30 | 8 | 0.3 | -5, 21 | 13 | 0.1 | -0.9, 26 |
|  | **5** | 0.03 | 0.7 | -0.11, 0.17 | 0.1 | 0.2 | -0.03, 0.16 | **0.1*** | **0.04** | **0.003, 0.2** |
|  | **6** | 11 | 0.4 | -14, 36 | 8 | 0.3 | -8, 25 | 10 | 0.2 | -6, 25 |
| **Lean mass index (kg/m^2^)** | **1** | -1 | 0.8 | -12, 10 | 4.6 | 0.3 | -3, 13 | 5 | 0.3 | -3, 12 |
|  | **2** | 0.01 | 0.9 | -0.08, 0.09 | 0.03 | 0.3 | -0.03, 0.09 | 0.04 | 0.2 | -0.02, 0.1 |
|  | **3** | 0.003 | 0.7 | -0.01, 0.01 | 0.01 | 0.2 | -0.004, 0.01 | 0.005 | 0.3 | -0.004, 0.01 |
|  | **4** | 5 | 0.1 | -0.7, 10 | **4*** | **0.04** | **0.2, 8** | **5*** | **0.03** | **0.58, 8.5** |
|  | **5** | 0.02 | 0.3 | -0.02, 0.06 | **0.03*** | **0.04** | **0.001, 0.06** | **0.03*** | **0.02** | **0.005, 0.06** |
|  | **6** | 1.9 | 0.6 | -5.16, 8.92 | 1.0 | 0.7 | -4.07, 6.06 | 1.2 | 0.6 | -3.9, 6.3 |
| **Fat mass (kg)** | **1** | 54 | 0.1 | 0.05, 108 | 36 | 0.1 | -6, 77 | 35 | 0.1 | -6, 77 |
|  | **2** | 0.1 | 0.5 | -0.3, 0.6 | 0.2 | 0.3 | -0.1, 0.5 | 0.3 | 0.1 | -0.02, 0.6 |
|  | **3** | 0.1 | 0.1 | -0.01, 0.1 | 0.05 | 0.1 | -0.001, 0.09 | 0.04 | 0.1 | -0.01, 0.09 |
|  | **4** | -11 | 0.5 | -39, 18 | 2 | 0.9 | -17, 20 | 7 | 0.5 | -13, 26 |
|  | **5** | -0.02 | 0.8 | -0.23, 0.18 | 0.005 | 0.9 | -0.13, 0.14 | 0.04 | 0.5 | -0.1, 0.19 |
|  | **6** | 6 | 0.7 | -29, 40 | 6 | 0.6 | -17, 30 | 8 | 0.5 | -14, 31 |
| **Fat mass index (kg/m^2^)** | **1** | 17 | 0.1 | -4, 37 | 9 | 0.2 | -6, 24 | 9 | 0.2 | -6, 24 |
|  | **2** | 0.04 | 0.7 | -0.12, 0.19 | 0.1 | 0.4 | -0.07, 0.16 | 0.1 | 0.2 | -0.04, 0.20 |
|  | **3** | 0.02 | 0.2 | -0.01, 0.04 | 0.01 | 0.2 | -0.01, 0.03 | 0.0 | 0.3 | -0.01, 0.03 |
|  | **4** | -4 | 0.4 | -16, 7 | 0.6 | 0.9 | -6.6, 7.7 | 2 | 0.6 | -5, 10 |
|  | **5** | -0.01 | 0.8 | -0.09, 0.07 | 0.0004 | 1.0 | -0.05, 0.05 | 0.01 | 0.7 | -0.04, 0.07 |
|  | **6** | 0.2 | 1.0 | -13.3, 13.7 | 0.5 | 0.9 | -8.3, 9.3 | 1 | 0.8 | -7, 10 |
| **% Fat mass** | **1** | **64*** | **0.05** | **0.2, 128** | 35 | 0.1 | -7, 76 | 34 | 0.1 | -7, 76 |
|  | **2** | 0.2 | 0.4 | -0.3, 0.7 | 0.2 | 0.2 | -0.1, 0.5 | 0.3 | 0.1 | -0.04, 0.6 |
|  | **3** | 0.1 | 0.1 | -0.02, 0.1 | 0.04 | 0.1 | -0.01, 0.09 | 0.04 | 0.1 | -0.01, 0.08 |
|  | **4** | -18 | 0.3 | -51, 14 | -5 | 0.6 | -21, 12 | -0.7 | 0.9 | -18, 17 |
|  | **5** | -0.04 | 0.7 | -0.28, 0.19 | -0.04 | 0.5 | -0.16, 0.08 | -0.01 | 0.9 | -0.13, 0.12 |
|  | **6** | -4 | 0.9 | -44, 37 | -0.9 | 0.9 | -21, 20 | 0.9 | 0.9 | -19, 21 |
| **Android fat mass (kg)** | **1** | 4 | 0.1 | -0.5, 9 | 3 | 0.1 | -0.8, 7 | 3 | 0.1 | -0.8, 7 |
|  | **2** | 0.01 | 0.6 | -0.03, 0.05 | 0.01 | 0.3 | -0.01, 0.04 | 0.02 | 0.1 | -0.01, 0.06 |
|  | **3** | 0.005 | 0.1 | -0.001, 0.01 | 0.004 | 0.1 | -0.0003, 0.01 | 0.004 | 0.1 | -0.001, 0.01 |
|  | **4** | -0.9 | 0.5 | -3, 2 | 0.2 | 0.8 | -1.6, 2 | 0.5 | 0.6 | -1, 2 |
|  | **5** | -0.003 | 0.8 | -0.02, 0.02 | -0.001 | 0.9 | -0.01, 0.01 | 0.003 | 0.7 | -0.01, 0.02 |
|  | **6** | 0.9 | 0.5 | -2., 4 | 0.9 | 0.4 | -1, 3 | 1.0 | 0.3 | -1, 3 |
| **% Android fat mass** | **1** | 70 | 0.1 | -2, 142 | 41 | 0.1 | -9, 92 | 41 | 0.1 | -9, 92 |
|  | **2** | 0.2 | 0.5 | -0.4, 0.7 | 0.2 | 0.3 | -0.2, 0.6 | 0.3 | 0.1 | -0.1, 0.7 |
|  | **3** | 0.1 | 0.1 | -0.02, 0.1 | 0.1 | 0.1 | -0.01, 0.1 | 0.0 | 0.1 | -0.01, 0.1 |
|  | **4** | -23 | 0.2 | -58, 14 | -7 | 0.5 | -28, 13 | -5 | 0.7 | -26, 17 |
|  | **5** | -0.5 | 0.8 | -5, 4 | -0.5 | 0.6 | -3, 2 | -0.03 | 0.7 | -0.19, 0.12 |
|  | **6** | 0.3 | 1.0 | -44, 45 | 2 | 0.9 | -25, 29 | 4 | 0.8 | -22, 30 |
| **Android-gynoid fat ratio (AG)** | **1** | -0.3 | 0.5 | -1.2, 0.6 | -0.3 | 0.6 | -1.2, 0.7 | -0.3 | 0.6 | -1.2, 0.7 |
|  | **2** | -0.005 | 0.2 | -0.01, 0.002 | -0.005 | 0.2 | -0.01, 0.002 | -0.005 | 0.2 | -0.01, 0.002 |
|  | **3** | -0.001 | 0.3 | -0.002, 0.001 | -0.0005 | 0.3 | -0.002, 0.001 | -0.001 | 0.3 | -0.002, 0.001 |
|  | **4** | **-0.5*** | **0.02** | **-0.8, -0.1** | **-0.4*** | **0.04** | **-0.83, -0.03** | -0.4 | 0.1 | -0.8, 0.1 |
|  | **5** | **-0.002*** | **0.04** | **-0.01, -0.0001** | -0.003 | 0.1 | -0.01, 0.00004 | -0.002 | 0.1 | -0.01, 0.001 |
|  | **6** | -0.1 | 0.8 | -0.6, 0.5 | -0.1 | 0.8 | -0.6, 0.4 | -0.03 | 0.9 | -0.54, 0.47 |
| **Fasting glucose (mmol/L)** | **1** | -2 | 0.3 | -5, 2 | -2 | 0.4 | -5, 2 | -2 | 0.4 | -5, 2 |
|  | **2** | -0.02 | 0.3 | -0.04, 0.01 | -0.01 | 0.3 | -0.04, 0.01 | -0.01 | 0.4 | -0.04, 0.01 |
|  | **3** | -0.001 | 0.7 | -0.004, 0.003 | 0.0005 | 0.8 | -0.004, 0.003 | -0.001 | 0.7 | -0.005, 0.003 |
|  | **4** | -1 | 0.1 | -3, 0.1 | -1.2 | 0.1 | -3, 0.2 | -1.2 | 0.1 | -3, 0.2 |
|  | **5** | -0.01 | 0.1 | -0.02, 0.001 | -0.01 | 0.1 | -0.02, 0.00 | 0.0 | 0.1 | -0.02, 0.003 |
|  | **6** | -0.3 | 0.7 | -2.3, 1.6 | -0.1 | 0.9 | -1.9, 1.6 | 0.1 | 0.9 | -1.7, 1.8 |
| **Fasting insulin (uIU/mL)** | **1** | -5 | 0.7 | -38, 29 | -8 | 0.6 | -34, 19 | -7.8 | 0.6 | -34, 19 |
|  | **2** | -0.1 | 0.4 | -0.3, 0.1 | -0.1 | 0.4 | -0.3, 0.1 | -0.1 | 0.6 | -0.3, 0.2 |
|  | **3** | -0.005 | 0.8 | -0.04, 0.03 | -0.01 | 0.7 | -0.04, 0.02 | -0.01 | 0.6 | -0.04, 0.02 |
|  | **4** | -3 | 0.6 | -15, 9 | -5 | 0.3 | -14, 5 | -4 | 0.4 | -14, 5 |
|  | **5** | -0.01 | 0.9 | -0.1, 0.08 | -0.04 | 0.3 | -0.11, 0.03 | -0.04 | 0.4 | -0.11, 0.04 |
|  | **6** | -9 | 0.2 | -23, 6 | -10 | 0.1 | -22, 2 | -10 | 0.1 | -23, 2 |
| **HOMA-IR** | **1** | -1 | 0.7 | -7, 5 | -2 | 0.6 | -7, 4 | -2 | 0.5 | -8, 4 |
|  | **2** | -0.02 | 0.3 | -0.07, 0.02 | -0.02 | 0.4 | -0.06, 0.02 | -0.02 | 0.5 | -0.06, 0.03 |
|  | **3** | -0.001 | 0.8 | -0.01, 0.01 | -0.001 | 0.7 | -0.01, 0.01 | -0.002 | 0.6 | -0.01, 0.005 |
|  | **4** | -0.8 | 0.5 | -3.3, 1.7 | -1 | 0.3 | -3, 0.8 | -1.0 | 0.3 | -3, 0.9 |
|  | **5** | -0.003 | 0.8 | -0.02, 0.02 | -0.01 | 0.2 | -0.02, 0.01 | -0.01 | 0.3 | -0.02, 0.01 |
|  | **6** | -2.0 | 0.2 | -5.4, 1.4 | -2.3 | 0.1 | -5.2, 0.5 | -2.3 | 0.1 | -5.2, 0.61 |
| **Low density lipoprotein (mmol/L)** | **1** | 0.8 | 0.8 | -4.2, 5.8 | 0.5 | 0.8 | -4.2, 5.3 | 0.7 | 0.8 | -4, 5.4 |
|  | **2** | 0.1 | 0.7 | -0.03, 0.05 | 0.01 | 0.7 | -0.03, 0.05 | -0.0005 | 1.0 | -0.04, 0.04 |
|  | **3** | 0.001 | 0.8 | -0.005, 0.01 | 0.001 | 0.9 | -0.005, 0.01 | -0.002 | 0.2 | -0.005, 0.001 |
|  | **4** | -0.4 | 0.7 | -2.4, 1.6 | -0.5 | 0.6 | -2.5, 1.5 | -0.5 | 0.7 | -2.6, 1.6 |
|  | **5** | -0.004 | 0.6 | -0.02, 0.01 | -0.01 | 0.4 | -0.02, 0.01 | -0.01 | 0.5 | -0.02, 0.01 |
|  | **6** | -0.3 | 0.8 | -2.6, 2.1 | -0.3 | 0.8 | -2.8, 2.2 | -0.3 | 0.8 | -3.0, 2.4 |
| **Triglycerides (mmol/L)** | **1** | -1.7 | 0.2 | -4.4, 1.06 | -1.8 | 0.2 | -4.5, 0.95 | -1.8 | 0.2 | -4.5, 1.0 |
|  | **2** | -0.02 | 0.1 | -0.04, 0.005 | -0.02 | 0.1 | -0.04, 0.005 | -0.02 | 0.1 | -0.04, 0.003 |
|  | **3** | -0.002 | 0.2 | -0.005, 0.001 | -0.002 | 0.2 | -0.01, 0.001 | -0.002 | 0.2 | -0.01, 0.001 |
|  | **4** | -0.4 | 0.3 | -1.1, 0.34 | -0.4 | 0.3 | -1.1, 0.33 | -0.3 | 0.4 | -1.04, 0.42 |
|  | **5** | -0.003 | 0.2 | -0.01, 0.002 | -0.003 | 0.3 | -0.01, 0.002 | -0.002 | 0.4 | -0.01, 0.003 |
|  | **6** | -0.6 | 0.2 | -1.4, 0.25 | -0.6 | 0.2 | -1.4, 0.26 | -0.5 | 0.2 | -1.4, 0.38 |

**^#^1- rehabilitation weight gain as ΔWAZ/day, 2 - rehabilitation weight gain as Δg/kg/day, 3 - rehabilitation weight gain as Δg/day, 4- post-recovery weight gain as ΔWAZ/month,**

**5 - post-recovery weight gain as Δg/kg/month, 6 - post-recovery height gain as ΔHAZ/month.  ^‘a‘^ Adjusted for age, sex and height for blood pressure only; ^‘b‘^ adjusted for age, sex, weight-for-age Z score at minimum weight and height for blood pressure only. * Statistical significance at *p* < 0.05.**
