# Supplementary material for: Faster rehabilitation weight gain during childhood is associated with risk of non-communicable disease in adult survivors of severe acute malnutrition
Source: PLOS Glob Public Health. 2023 Dec 21;3(12):e0002698. doi: 10.1371/journal.pgph.0002698 (PMC10734994; doi:10.1371/journal.pgph.0002698)
Supplement: S2 Table — (DOCX) [file pgph.0002698.s002.docx]

**Supplementary Table 2:** Results of quintile grouped linear regressions of rehabilitation weight gain and post-recovery weight and height gain against NCD risk indicators in 273 adult survivors of severe acute malnutrition.

| **NCD indicator** | **Weight/**  **height**  **Gain**  **Definition^#^** | **Unadjusted coefficient** | ***p*-value** | **95% CI** | **Adjusted coefficient ^a^** | ***p*-value** | **95% CI** | **Adjusted**  **coefficient ^b^** | ***p*-value** | **95% CI** |
| --- | --- | --- | --- | --- | --- | --- | --- | --- | --- | --- |
| **Systolic BP (mmHg)** | **1** | -0.1 | 0.8 | -1.15, 0.91 | -0.1 | 0.9 | -1.09, 0.90 | -0.1 | 0.8 | -1.1, 0.86 |
|  | **2** | -0.3 | 0.6 | -1.34, 0.72 | -0.1 | 0.9 | -1.07, 0.90 | -0.4 | 0.5 | -1.4, 0.65 |
|  | **3** | -0.4 | 0.5 | -1.40, 0.65 | -0.5 | 0.3 | -1.48, 0.52 | -0.4 | 0.5 | -1.4, 0.62 |
|  | **4** | -0.5 | 0.6 | -2.60, 1.55 | -0.6 | 0.6 | -2.69, 1.49 | -0.9 | 0.4 | -3.1, 1.3 |
|  | **5** | -0.9 | 0.4 | -3.00, 1.13 | -0.7 | 0.5 | -2.81, 1.37 | -1.0 | 0.4 | -3.2, 1.2 |
|  | **6** | -0.4 | 0.8 | -2.86, 2.13 | -0.4 | 0.8 | -3.02, 2.24 | -0.3 | 0.8 | -3.0, 2.3 |
| **Diastolic BP (mmHg)** | **1** | 0.2 | 0.7 | -0.78, 1.14 | 0.2 | 0.7 | -0.74, 1.11 | 0.2 | 0.7 | -0.76, 1.1 |
|  | **2** | 0.0 | 0.9 | -0.92, 1.00 | 0.2 | 0.3 | -0.19, 0.55 | 0.05 | 0.9 | -0.91, 1.0 |
|  | **3** | 0.1 | 0.8 | -0.83, 1.09 | 0.1 | 0.9 | -0.87, 0.99 | 0.2 | 0.7 | -0.8, 1.1 |
|  | **4** | -0.6 | 0.5 | -2.52, 1.28 | -1.2 | 0.9 | -26.64, 24.34 | -3 | 0.8 | -30, 24 |
|  | **5** | -1.0 | 0.3 | -2.87, 0.92 | -0.4 | 0.7 | -2.29, 1.44 | -0.5 | 0.6 | -2.5, 1.4 |
|  | **6** | 0.2 | 0.9 | -2.26, 2.56 | 0.6 | 0.6 | -1.8, 3.0 | 0.7 | 0.6 | -1.7, 3.1 |
| **BMI (kg/m^2^)** | **1** | 0.4 | 0.1 | -0.01, 0.9 | 0.4 | 0.04 | 0.02, 0.8 | 0.4 | 0.04 | 0.02, 0.79 |
|  | **2** | 0.3 | 0.3 | -0.2, 0.7 | 0.4 | 0.1 | -0.04, 0.74 | 0.5 | 0.02 | 0.09, 0.9 |
|  | **3** | 0.4 | 0.1 | -0.04, 0.8 | 0.4 | 0.1 | -0.02, 0.8 | 0.3 | 0.1 | -0.08, 0.7 |
|  | **4** | -0.2 | 0.7 | -1.1, 0.72 | 0.3 | 0.5 | -0.47, 1.0 | 0.4 | 0.3 | -0.4, 1.1 |
|  | **5** | -0.1 | 0.8 | -1.0, 0.8 | 0.2 | 0.6 | -0.5, 1.0 | 0.4 | 0.3 | -0.4, 1.1 |
|  | **6** | -0.1 | 0.8 | -1.3, 1.0 | 0.1 | 0.9 | -1.0, 1.0 | 0.1 | 0.9 | -1.0, 1.0 |
|  | **1** | **1.2*** | **0.03** | **0.13, 2.2** | **1.2*** | **0.01** | **0.3, 2.2** | **1.2*** | **0.01** | **0.3, 2.1** |
|  | **2** | 0.6 | 0.3 | -0.5, 1.6 | 0.9 | 0.06 | -0.05, 1.8 | **1.4*** | **0.005** | **0.43, 2.4** |
|  | **3** | **1.4*** | **0.01** | **0.3, 2.4** | **1.4*** | **0.004** | **0.43, 2.3** | **1.2*** | **0.01** | **0.24, 2.1** |
| **Waist circumference (cm)** | **4** | -1 | 0.3 | -3, 1 | 0.1 | 0.9 | -1.7, 1.9 | 0.3 | 0.8 | -1.6, 2.2 |
|  | **5** | -1 | 0.3 | -3, 1 | -0.1 | 0.9 | -1.9, 1.7 | 0.2 | 0.8 | -1.7, 2.1 |
|  | **6** | 0.5 | 0.7 | -2.3, 3.2 | 0.9 | 0.5 | -1.5, 3.2 | 0.9 | 0.5 | -1.5, 3.2 |
| **Waist:hip ratio** | **1** | 0.002 | 0.5 | -0.004, 0.01 | 0.003 | 0.2 | -0.002, 0.01 | 0.003 | 0.2 | -0.002, 0.01 |
|  | **2** | 0.0002 | 0.9 | -0.01, 0.01 | 0.002 | 0.4 | -0.003, 0.01 | 0.002 | 0.4 | -0.003, 0.01 |
|  | **3** | 0.003 | 0.3 | -0.003, 0.01 | 0.004 | 0.2 | -0.001, 0.01 | 0.004 | 0.1 | -0.001, 0.01 |
|  | **4** | -0.002 | 0.7 | -0.01, 0.01 | -0.004 | 0.7 | -0.01, 0.01 | -0.004 | 0.5 | -0.02, 0.01 |
|  | **5** | -0.01 | 0.4 | -0.02, 0.01 | -0.002 | 0.6 | -0.01, 0.01 | -0.01 | 0.4 | -0.02, 0.01 |
|  | **6** | 0.003 | 0.7 | -0.01, 0.02 | 0.002 | 0.8 | -0.01, 0.01 | 0.002 | 0.8 | -0.01, 0.01 |
| **Lean mass (kg)** | **1** | 0.3 | 0.5 | -0.59, 1.17 | **0.8*** | **0.01** | **0.16, 1.39** | **0.78*** | **0.02** | **0.17, 1.4** |
|  | **2** | 0.1 | 0.8 | -0.77, 0.98 | 0.4 | 0.3 | -0.28, 0.97 | **0.74*** | **0.02** | **0.1, 1.4** |
|  | **3** | 0.5 | 0.3 | -0.41, 1.34 | **0.9*** | **0.004** | **0.28, 1.50** | **0.7*** | **0.02** | **0.1, 1.3** |
|  | **4** | 1.2 | 0.1 | -0.35, 2.74 | 0.7 | 0.2 | -0.39, 1.71 | 0.95 | 0.08 | -0.1, 2 |
|  | **5** | 0.6 | 0.5 | -0.97, 2.15 | 0.9 | 0.1 | -0.16, 1.93 | **1.3*** | **0.02** | **0.27, 2.4** |
|  | **6** | 1.4 | 0.2 | -0.69, 3.39 | 0.7 | 0.3 | -0.64, 2.07 | 0.7 | 0.3 | -0.6, 2 |
| **Lean mass index (kg/m^2^)** | **1** | 0.02 | 0.9 | -0.20, 0.24 | 0.14 | 0.08 | -0.02, 0.30 | 0.14 | 0.09 | -0.02, 0.3 |
|  | **2** | 0.04 | 0.8 | -0.18, 0.25 | 0.11 | 0.181 | -0.05, 0.27 | 0.14 | 0.09 | -0.02, 0.3 |
|  | **3** | 0.02 | 0.9 | -0.20, 0.24 | 0.12 | 0.124 | -0.03, 0.28 | 0.1 | 0.1 | -0.05, 0.3 |
|  | **4** | **0.45*** | **0.03** | **0.04, 0.86** | **0.34*** | **0.03** | **0.03, 0.65** | **0.35*** | **0.03** | **0.04, 0.7** |
|  | **5** | 0.28 | 0.2 | -0.14, 0.69 | **0.38*** | **0.02** | **0.07, 0.69** | **0.43*** | **0.008** | **0.1, 0.7** |
|  | **6** | 0.24 | 0.4 | -0.34, 0.83 | 0.08 | 0.7 | -0.34, 0.50 | 0.08 | 0.7 | -0.3, 0.5 |
| **Fat mass (kg)** | **1** | **1.17*** | **0.03** | **0.09, 2.26** | **0.8*** | **0.05** | **0.004, 1.7** | **0.8*** | **0.05** | **0.01, 1.7** |
|  | **2** | 0.7 | 0.2 | -0.43, 1.75 | 0.7 | 0.1 | -0.1, 1.6 | **1.1*** | **0.01** | **0.23, 2** |
|  | **3** | **1.15*** | **0.04** | **0.06, 2.23** | 0.8 | 0.1 | -0.003, 1.67 | 0.7 | 0.1 | -0.1, 1.5 |
|  | **4** | -1.5 | 0.2 | -3.75, 0.72 | -0.2 | 0.8 | -1.70, 1.32 | 0.1 | 0.9 | -1.4, 1.7 |
|  | **5** | -0.9 | 0.4 | -3.14, 1.37 | -0.3 | 0.7 | -1.86, 1.17 | 0.05 | 0.9 | -1.5, 1.6 |
|  | **6** | -0.5 | 0.7 | -3.33, 2.41 | 0.4 | 0.7 | -1.62, 2.33 | 0.4 | 0.7 | -1.5, 2.3 |
| **Fat mass index (kg/m^2^)** | **1** | 0.4 | 0.1 | -0.04, 0.8 | 0.2 | 0.1 | -0.07, 0.5 | 0.23 | 0.1 | -0.07, 0.54 |
|  | **2** | 0.2 | 0.4 | -0.2, 0.6 | 0.2 | 0.2 | -0.09, 0.5 | 0.32 | 0.05 | -0.0001, 0.6 |
|  | **3** | 0.3 | 0.1 | -0.07, 0.8 | 0.2 | 0.2 | -0.09, 0.52 | 0.2 | 0.3 | -0.1, 0.5 |
|  | **4** | -0.6 | 0.2 | -1.5, 0.3 | -0.06 | 0.8 | -0.63, 0.51 | 0.03 | 0.9 | -0.6, 0.6 |
|  | **5** | -0.4 | 0.4 | -1.3, 0.5 | -0.2 | 0.6 | -0.7, 0.4 | -0.03 | 0.9 | -0.6, 0.6 |
|  | **6** | -0.3 | 0.6 | -1.4, 0.8 | 0.001 | 1.0 | -0.73, 0.74 | 0.001 | 1 | -0.72, 0.72 |
| **% Fat mass** | **1** | **1.4*** | **0.03** | **0.13, 2.69** | **0.85*** | **0.04** | **0.02, 1.68** | **0.85*** | **0.04** | **0.02, 1.7** |
|  | **2** | 0.9 | 0.2 | -0.42, 2.14 | 0.8 | 0.1 | -0.19, 2.35 | **1.1*** | **0.02** | **0.21, 1.9** |
|  | **3** | 1.3 | 0.1 | -0.03, 2.53 | 0.7 | 0.1 | -0.09, 1.57 | 0.7 | 0.1 | -0.2, 1.5 |
|  | **4** | -2.1 | 0.1 | -4.62, 0.50 | -0.4 | 0.5 | -1.77, 0.88 | -0.2 | 0.8 | -1.6, 1.2 |
|  | **5** | -1.1 | 0.4 | -3.64, 1.54 | -0.7 | 0.3 | -1.98, 0.67 | -0.03 | 0.6 | -1.8, 1.0 |
|  | **6** | -1.4 | 0.4 | -4.72, 2.02 | -0.2 | 0.8 | -1.94, 1.55 | -0.2 | 0.8 | -1.9, 1.5 |
| **Android fat mass (kg)** | **1** | 0.09 | 0.1 | -0.01, 0.19 | 0.07 | 0.1 | -0.01, 0.15 | 0.07 | 0.1 | -0.01, 0.15 |
|  | **2** | 0.05 | 0.4 | -0.05, 0.14 | 0.06 | 0.1 | -0.02, 0.14 | **0.09*** | **0.03** | **0.01, 0.17** |
|  | **3** | 0.09 | 0.1 | -0.01, 0.18 | 0.07 | 0.1 | -0.01, 0.15 | 0.06 | 0.2 | -0.02, 0.1 |
|  | **4** | -0.13 | 0.2 | -0.33, 0.06 | -0.02 | 0.8 | -0.16, 0.13 | -0.002 | 0.9 | -0.15, 0.14 |
|  | **5** | -0.09 | 0.3 | -0.29, 0.10 | -0.03 | 0.6 | -0.18, 0.11 | -0.01 | 0.9 | -0.2, 0.1 |
|  | **6** | 0.01 | 1.0 | -0.24, 0.26 | 0.07 | 0.5 | -0.12, 0.25 | 0.06 | 0.5 | -0.1, 0.2 |
| **% Android fat mass** | **1** | **1.5*** | **0.04** | **0.07, 3** | 0.98 | 0.06 | -0.04, 1.99 | 0.98 | 0.06 | -0.04, 2 |
|  | **2** | 0.9 | 0.3 | -0.61, 2.30 | 0.9 | 0.09 | -0.12, 1.91 | **1.2*** | **0.03** | **0.12, 2.2** |
|  | **3** | 1.4 | 0.1 | -0.06, 2.84 | 0.9 | 0.08 | -0.12, 1.91 | 0.8 | 0.1 | -0.2, 1.8 |
|  | **4** | -2.4 | 0.1 | -5.22, 0.48 | -0.6 | 0.5 | -2.25, 1.04 | -0.4 | 0.6 | -2.2, 1.3 |
|  | **5** | -1.3 | 0.4 | -4, 2 | -0.8 | 0.3 | -2.48, 0.81 | -0.6 | 0.5 | -2.4, 1.1 |
|  | **6** | -1.1 | 0.6 | -4.76, 2.65 | 0.1 | 0.9 | -2.12, 2.33 | 0.1 | 0.9 | -2.1, 2.3 |
| **Android-gynoid fat ratio (AG)** | **1** | -0.01 | 0.3 | -0.03, 0.01 | -0.01 | 0.3 | -0.02, 0.01 | -0.01 | 0.3 | -0.03, 0.01 |
|  | **2** | **-0.02*** | **0.05** | **-0.04, -0.0004** | -0.02 | 0.6 | -0.04, 0.001 | -0.02 | 0.06 | -0.04, 0.001 |
|  | **3** | -0.01 | 0.2 | -0.03, 0.01 | -0.01 | 0.2 | -0.03, 0.01 | -0.01 | 0.2 | -0.03, 0.01 |
|  | **4** | **-0.04*** | **0.02** | **-0.07, -0.01** | **-0.04*** | **0.02** | **-0.07, -0.005** | -0.03 | 0.07 | -0.06, 0.002 |
|  | **5** | **-0.04*** | **0.01** | **-0.07, -0.01** | **-0.04*** | **0.02** | **-0.07, -0.008** | **-0.03*** | **0.05** | **-0.07, -0.001** |
|  | **6** | 0.01 | 0.8 | -0.04, 0.5 | 0.003 | 0.9 | -0.04, 0.05 | 0.003 | 0.9 | -0.04, 0.04 |
| **Fasting glucose (mmol/L)** | **1** | -0.05 | 0.2 | -0.11, 0.02 | -0.04 | 0.2 | -0.11, 0.03 | -0.04 | 0.2 | -0.11, 0.03 |
|  | **2** | -0.06 | 0.1 | -0.12, 0.01 | -0.05 | 0.1 | -0.117, 0.01 | -0.05 | 0.2 | -0.12, 0.02 |
|  | **3** | -0.04 | 0.2 | -0.11, 0.03 | -0.03 | 0.3 | -0.10, 0.03 | -0.04 | 0.3 | -0.11, 0.03 |
|  | **4** | -0.07 | 0.1 | -0.18, 0.03 | -0.06 | 0.2 | -0.16, 0.04 | -0.06 | 0.2 | -0.16, 0.04 |
|  | **5** | -0.09 | 0.1 | -0.18, 0.00 | -0.07 | 0.1 | -0.16, 0.02 | -0.07 | 0.2 | -0.17, 0.03 |
|  | **6** | -0.04 | 0.6 | -0.19, 0.11 | -0.003 | 1.0 | -0.14, 0.13 | 0.01 | 0.9 | -0.12, 0.145 |
| **Fasting insulin (uIU/mL)** | **1** | -0.1 | 0.8 | -0.65, 0.49 | -0.2 | 0.5 | -0.70, 0.34 | -0.2 | 0.5 | -0.7, 0.35 |
|  | **2** | -0.3 | 0.3 | -0.84, 0.26 | -0.3 | 0.3 | -0.78, 0.23 | -0.2 | 0.4 | -0.8, 0.3 |
|  | **3** | -0.2 | 0.6 | -0.71, 0.42 | -0.3 | 0.3 | -0.79, 0.24 | -0.3 | 0.2 | -0.8, 0.2 |
|  | **4** | -0.2 | 0.7 | -1.03, 0.68 | -0.4 | 0.2 | -1.07, 0.27 | -0.4 | 0.3 | -1.1, 0.29 |
|  | **5** | -0.1 | 0.8 | -0.90, 0.70 | -0.4 | 0.2 | -1.06, 0.21 | -0.4 | 0.2 | -1.1, 0.25 |
|  | **6** | -0.6 | 0.3 | -1.79, 0.54 | -0.8 | 0.1 | -1.77, 0.14 | -0.8 | 0.1 | -1.8, 0.2 |
| **HOMA-IR** | **1** | -0.02 | 0.7 | -0.1, 0.1 | -0.04 | 0.5 | -0.15, 0.07 | -0.04 | 0.5 | -0.15, 0.07 |
|  | **2** | -0.07 | 0.2 | -0.2, 0.05 | 0.07 | 0.2 | -0.2, 0.04 | -0.06 | 0.3 | -0.2, 0.05 |
|  | **3** | -0.04 | 0.5 | -0.2, 0.1 | -0.06 | 0.3 | -0.2, 0.05 | -0.07 | 0.2 | -0.2, 0.04 |
|  | **4** | -0.04 | 0.6 | -0.2, 0.1 | -0.09 | 0.2 | -0.2, 0.05 | -0.08 | 0.2 | -0.22, 0.05 |
|  | **5** | -0.04 | 0.6 | -0.2, 0.1 | -0.1 | 0.1 | -0.2, 0.03 | -0.09 | 0.2 | -0.2, 0.04 |
|  | **6** | -0.2 | 0.2 | -0.42, 0.11 | -0.2 | 0.09 | -0.41, 0.03 | -0.2 | 0.1 | -0.42, 0.04 |
| **Low density lipoprotein (mmol/L)** | **1** | 0.03 | 0.5 | -0.07, 0.1 | 0.01 | 0.8 | -0.09, 0.1 | 0.01 | 0.8 | -0.08, 0.11 |
|  | **2** | 0.02 | 0.6 | -0.07, 0.1 | 0.02 | 0.7 | -0.08, 0.1 | -0.01 | 0.9 | -0.1, 0.1 |
|  | **3** | 0.01 | 0.8 | -0.1, 0.1 | -0.01 | 0.9 | -0.1, 0.1 | 0.01 | 0.9 | -0.1, 0.1 |
|  | **4** | 0.07 | 0.4 | -0.1, 0.2 | 0.06 | 0.5 | -0.1, 0.2 | 0.07 | 0.4 | -0.1, 0.2 |
|  | **5** | 0.05 | 0.5 | -0.1, 0.2 | 0.04 | 0.6 | 0.1, 0.2 | 0.05 | 0.5 | -0.1, 0.2 |
|  | **6** | 0.09 | 0.4 | -0.14, 0.32 | 0.1 | 0.4 | -0.15, 0.35 | 0.1 | 0.4 | 0.2, 0.4 |
| **Triglycerides (mmol/L)** | **1** | -0.02 | 0.4 | -0.08, 0.04 | -0.03 | 0.4 | -0.08, 0.03 | -0.03 | 0.4 | -0.08, 0.03 |
|  | **2** | -0.03 | 0.3 | -0.09, 0.02 | -0.03 | 0.3 | -0.09, 0.02 | -0.04 | 0.2 | -0.1, 0.02 |
|  | **3** | -0.03 | 0.3 | -0.1, 0.03 | -0.03 | 0.3 | -0.1, 0.03 | -0.03 | 0.3 | -0.1, 0.03 |
|  | **4** | -0.03 | 0.4 | -0.09, 0.03 | -0.03 | 0.3 | -0.09, 0.03 | -0.02 | 0.4 | -0.09, 0.04 |
|  | **5** | -0.05 | 0.1 | -0.1, 0.01 | -0.04 | 0.2 | -0.1, 0.02 | -0.03 | 0.2 | -0.1, 0.03 |
|  | **6** | -0.07 | 0.1 | -0.2, 0.01 | -0.06 | 0.1 | -0.1, 0.02 | -0.05 | 0.2 | -0.1, 0.03 |

**^#^1- rehabilitation weight gain as ΔWAZ/day, 2 - rehabilitation weight gain as Δg/kg/day, 3 - rehabilitation weight gain as Δg/day, 4- post-recovery weight gain as ΔWAZ/month,**

**5 - post-recovery weight gain as Δg/kg/month, 6 - post-recovery height gain as ΔHAZ/month.  ^‘a‘^ Adjusted for age, sex and height for blood pressure only; ^‘b‘^ adjusted for age, sex, weight-for-age Z score at minimum weight and height for blood pressure only. * Statistical significance at *p* < 0.05.**
