# Supplementary material for: Faster rehabilitation weight gain during childhood is associated with risk of non-communicable disease in adult survivors of severe acute malnutrition
Source: PLOS Glob Public Health. 2023 Dec 21;3(12):e0002698. doi: 10.1371/journal.pgph.0002698 (PMC10734994; doi:10.1371/journal.pgph.0002698)
Supplement: Attachment — Submitted filename: Response to reviewers.docx [file pgph.0002698.s004.docx]

Journal Requirements:

1. Please review your reference list to ensure that it is complete and correct. If you have cited papers that have been retracted, please include the rationale for doing so in the manuscript text or remove these references and replace them with relevant current references. Any changes to the reference list should be mentioned in the rebuttal letter that accompanies your revised manuscript. If you need to cite a retracted article, indicate the article’s retracted status in the References list and also include a citation and full reference for the retraction notice.

**RESPONSE: The references were checked and appear correct.**

2. We noticed that you used "data not shown" in the manuscript. We do not allow these references, as the PLOS data access policy requires that all data be either published with the manuscript or made available in a publicly accessible database. Please amend the supplementary material to include the referenced data or remove the references.

**RESPONSE:** “Data not shown” has been removed from the manuscript and all (supporting) data are either published with the manuscript or available in a publicly accessible database

3. Please provide separate figure files in .tif or .eps format only and remove any figures embedded in your manuscript file. Please also ensure all files are under our size limit of 10MB.

For more information about figure files please see our guidelines:

https://journals.plos.org/globalpublichealth/s/figures 
<https://journals.plos.org/globalpublichealth/s/figures#loc-file-requirement>

**RESPONSE:** Four (4) separate figure files (.tif) have been uploaded.

4. We have noticed that you have a list of Supporting Information legends in your manuscript. However, there are no corresponding files uploaded to the submission. Please upload them as separate files with the item type 'Supporting Information.

**RESPONSE:** Two (2) supplementary tables have been uploaded as Supporting Information

**Reviewer #1:**

Thank you for taking the time to review our submission. Your specific concerns are addressed individually below.

1) Line 261, 262 need to mention the direction of the relationship would bring more clarity.

**RESPONSE:** **These suggested changes were made**

2) Supplementary table 1 – marking/highlighting the statistically significant relationship would be helpful in all tables

**RESPONSE:** This suggested change was made

3) Line 271 to 275 – specifying the direction of the association will bring clarity in understanding

**RESPONSE:** These suggested changes were made

4) In these participants the weight gain could be of 2 types; 1) weight gain due to increase in lean mass, 2) weight gain due increase in fat mass. This aspect needs to be looked into separately, as in, the participants who have gained weight beyond the threshold was there a difference in the composition of the weight gain (whether lean mass was gained or fat mass was gained) and if so, were the relationship to NCD risk differ. These being complex interactions there is a need to look into these interactions in depth.

**RESPONSE:** This is a fair question. However, it would be difficult to evaluate this without knowing what body composition was at the time of admission for SAM or post-recovery. We have therefore cited this as a limitation (lines 483-484). We have unpublished data from a group of Jamaican children who were hospitalized for SAM and fed enriched diets to promote rapid catch-up-growth (*Ann Nutr Metab* (2013) 63 (Suppl. 1): page 1517). The data suggest that, at recovery, lean mass (determined using deuterium dilution) in these children was consistent with reference values (71- 92% of body weight) and it is to be noted that some of these children experienced weight gain >10g/kg/day. However, as the children were still young, there was no data available on NCD risk in this group.

5) As already mentioned by the authors in the limitation post recovery factors are very important and would be confounding to all the findings, hence need to be looked into. The present findings do highlight the point that there is a need to be cautious about the rehabilitation and post recovery weight gain but many of the important confounding factors like diet, activity and environment have not been looked into which might affect the results differently.

**RESPONSE:** We agree with this point. In addition to citing this as a limitation, we have further clarified in the manuscript that data on potential confounders such as diet, activity and environment were **not available** for evaluation.

**Reviewer #2:**

The manuscript is well written but there are things that can be improved before acceptance. Please refer to the attached file for my suggested edits and their corresponding highlighted sections in the manuscript.

**RESPONSE:** Thank you for taking the time to review our submission. Your very detailed edits and comments are very much appreciated and will certainly improve the quality of the manuscript. All (except one) of your suggestions have been incorporated into the manuscript and are evident in the track changes. Please note that the abbreviation for weight-for-age z-scores (WAZ) was not introduced in the abstract as the term was not repeated. In line 59 weight-for-**height** z-scores was mentioned.
